# Motives for Crafting Work and Leisure: Focus on Opportunities at Work and Psychological Needs as Drivers of Crafting Efforts

**DOI:** 10.3390/ijerph182312294

**Published:** 2021-11-23

**Authors:** Merly Kosenkranius, Floor Rink, Miika Kujanpää, Jessica de Bloom

**Affiliations:** 1 Department of HRM & OB, Faculty of Economics and Business, University of Groningen, Nettelbosje 2, 9747 AE Groningen, The Netherlands; f.a.rink@rug.nl (F.R.); j.de.bloom@rug.nl (J.d.B.); 2 Faculty of Social Sciences (Psychology), Tampere University, Kalevantie 5, 33100 Tampere, Finland; Miika.Kujanpaa@usn.no; 3 School of Business, University of South-Eastern Norway, Bredalsveien 14, 3511 Hønefoss, Norway

**Keywords:** focus on opportunities at work, psychological needs, job crafting, off-job crafting

## Abstract

Employees of all ages can proactively shape their behavior to manage modern work–life challenges more effectively and this is known as crafting. Our goal is to better understand employees’ motives for engaging in crafting efforts in different life domains to fulfil their psychological needs. In a survey study with two measurement waves, we examined whether “focus on opportunities at work” (FoO)—the extent to which employees believe in new goals and opportunities in their occupational future—and psychological needs (i.e., approach and avoidance needs)—predicted crafting efforts at work and outside work (i.e., job and off-job crafting). Our hypotheses were largely confirmed in a study on 346 Finnish workers. Greater FoO led to greater approach needs (i.e., mastery, meaning, affiliation), which in turn explained higher engagement in both job and off-job crafting. Avoidance needs (i.e., detachment, relaxation) resulted in increased crafting efforts in both life domains directly. Our findings underline the importance of FoO for crafting efforts across life domains, and explain why this is the case (i.e., it activates approach-oriented psychological needs). By supporting workers in shifting their focus onto their future opportunities (regardless of their age), organizations can create environments conducive to crafting and ultimately sustainable work lives.

## 1. Introduction

Working life has changed dramatically over the last decade. With the retirement age rising, the workforce has become more age-diverse, and due to flexible work arrangements, work has also become more deregulated [1]. The ongoing COVID-19 pandemic and the accompanying shifts to remote work have further accelerated these societal trends [2]. As a result, employees are expected to adapt quickly to change [3], to work independently [4] and to manage their own careers proactively [5,6] for a longer period of time during their lifespan.

In order to keep workers healthy, engaged, and productive under these changing circumstances, organizations are keen to help employees to strike a sustainable work–life balance [7,8]. Research consistently demonstrates that job crafting may constitute a good strategy that employees can use to work effectively while also retaining their well-being over time. Job crafting and the rise in research on this phenomenon mirrors the heightened responsibility for self-management. Defined as employees’ proactive ways to shape their work to meet their personal needs and abilities [9,10], job crafters make self-initiated changes within their work environments. This approach to work promotes positive outcomes for employees and organizations such as higher work engagement [11] and job performance [12]. It is therefore important to further our understanding of why some people craft while others do not. Insights on the individual differences possibly underlying this strategy will give practitioners concrete guidelines on how to motivate employees to become more proactive crafters of their own work lives. This will be the focus in this paper.

Importantly, research so far has focused mainly on crafting efforts occurring in the job domain. Research on crafting outside the work domain is scarce. We address this gap in the literature by applying the Identity-Based Integrative Needs Model of Crafting (hereinafter, Needs Model of Crafting) [13], which portrays crafting as an holistic approach to life which spans both work and off-job time. In this new model, psychological needs, such as the need to do something meaningful or to feel connected to others, constitute both drivers and outcomes of people’s crafting efforts. Needs theories have described psychological needs as nutrients required to experience psychological growth and well-being [14]. Importantly, these psychological needs are not considered domain-specific but universal across life domains. Accordingly, the Needs Model of Crafting proposes that people will strive to satisfy their needs in different life domains by potentially engaging in both job crafting and off-job crafting efforts. The model assumes that during off-job, or leisure time, employees will also actively engage in crafting because they are then less burdened by work demands, yet benefit from it through spillover processes [13,15]. 

While every employee is capable of crafting and there is growing evidence of the beneficial effects of employees’ crafting efforts, there is individual variation in the extent to which people engage in crafting efforts in different life domains that is not yet well understood [16]. We aim to address this gap by investigating the combination of two important motivational antecedents of employees crafting efforts: focus on opportunities at work and psychological needs. First, we propose that employees’ crafting efforts are motivated by their focus on future opportunities at work—employees’ perceptions of their future professional opportunities (FoO) [17]. We derive this proposition from socioemotional selectivity theory [18,19], which claims that the time and opportunities people perceive they have left in life determine the goals they select and the behaviors they engage in to reach these goals. The subjectively experienced remaining time and opportunities for development of one’s career explain variance in motivation and behavior beyond chronological age. This theory thus predicts that a person’s FoO should affect crafting efforts in addition to age [16] and proactivity-related personality traits [20]. Moreover, given that employees’ FoO at work motivates their overall future goal selection and generally triggers proactive behaviors, it can be expected that FoO at work acts as a key motivator of both job and off-job crafting efforts. 

Second, despite psychological needs being universal across people and domains, there is variation in the importance that employees attach to different psychological needs [21]. In testing our reasoning, we rely on six key psychological needs specified in the crafting literature (i.e., detachment, relaxation, autonomy, mastery, meaning, and affiliation; see also the DRAMMA model) [22]. Each psychological need is expected to enhance employees’ engagement in crafting efforts that fulfil this need. For example, an employee who values close connections with co-workers could engage in job crafting for affiliation by organizing a weekly coffee break with colleagues, while employees with a strong need to experience meaning in their life might engage in off-job crafting by volunteering for a cause that is important to them. Thus, when combining socioemotional selectivity theory with the Needs Model of Crafting, it can be expected that employees with a high FoO (i.e., who have a more open-ended future perspective on their work) should particularly be driven by approach-oriented needs for future growth and development opportunities at work, which, in turn, should steer their crafting efforts to pursue these goals. These employees should thus develop weaker avoidance needs and engage less in crafting efforts to reduce or prevent work demands. 

Summing up, we focus (1) on employees’ focus on opportunities at work which may lead to enhanced crafting efforts both directly and via employees’ psychological needs, and (2) on life-domain overarching psychological needs which motivate crafting efforts in both work and non-work domains. To answer our research questions, we utilized the first two measurements of a two-wave survey of a large sample of Finnish employees with three measurements and three-month time lags between measurements. 

This study contributes to the existing literature in two ways. Firstly, we integrate life span research (more specifically: the role of “focus on opportunities at work”) and need theories to predict crafting efforts. We make optimal use of the time lags in our dataset and control for chronological age and proactive personality to demonstrate that, in addition to proactive crafting efforts being dependent on an individual’s age and personality, crafting may be influenced by more flexible motivational drivers: FoO at work and psychological needs. Secondly, we contribute to the crafting literature by recognizing that in addition to crafting their jobs, employees can craft their non-work time to gain new resources. Accordingly, we examine several key propositions of the recently developed Needs Model of Crafting [13]. Specifically, we aim to expand knowledge about psychological needs as crafting motives by testing approach (i.e., autonomy, mastery, meaning, and affiliation) and avoidance needs (detachment and relaxation) as motives for need-congruent crafting efforts (i.e., matching need and crafting effort). Taken together, the study insights will inform theorizing about the intrapersonal motivational drivers behind crafting. Finally, from a practical point of view, we further the understanding of why some people craft and others do not. In this way, practitioners can find better ways to motivate employees to engage in needs-satisfying crafting efforts.

## 2. Theory and Hypotheses

### 2.1. Crafting One’s Job in Accordance with Psychological Needs: Needs as Crafting Drivers

Instead of being passive adapters to circumstances, employees may engage in self-initiated behaviors to actively create more favorable future circumstances and to pursue self-concordant goals [6,20,23]. One way to do so is through job crafting, a concept defined as “the physical and cognitive changes that individuals make in the task or relational boundaries of their work” to shape their work identity and create more meaningful work for themselves [10]. 

Building on and extending Berg and colleagues’ [24] notion that employees find meaning via job crafting, Kooij and colleagues [25] found that crafting which focuses on employees’ motives, needs and values is positively linked to higher work engagement and job performance [26], and the absorption and dedication dimensions of work engagement [27]. In further support of this perspective, Bindl and colleagues [28] investigated individuals’ psychological needs in job crafting and showed that the strength of employees’ need for autonomy, competence and relatedness predicted different job crafting behaviors. 

Building on this earlier work, de Bloom and colleagues [13] recently developed the Needs Model of Crafting, which proposes that psychological needs are so fundamental as to transcend different life domains and constitute both motivators and outcomes of the crafting process (i.e., needs satisfaction). Furthermore, the model distinguishes between approach- and avoidance-focused needs and matching crafting efforts to satisfy given psychological needs. Avoidance-focused crafting is motivated by employees’ desire to reduce the negative aspects related to work or non-work time (e.g., crafting for relaxation), while approach-focused crafting refers to increasing the positive aspects of work or non-work time (e.g., crafting for meaning) [13,29,30]. 

The set of psychological needs which drives crafting efforts across life domains can be derived from the DRAMMA model [22]. The model proposes six needs having the potential to contribute to employees’ optimal functioning [31]: detachment, relaxation, autonomy, mastery, meaning and affiliation. Detachment from work refers to the process of both physically and mentally disengaging from all work-related matters, while relaxation is characterized by low mental or physical activation levels and little physical, social or intellectual effort [32]. Of the six dimensions, detachment from work and relaxation have been referred to as recovery experiences conducive to reducing perceived demands and psychobiological recovery processes, allowing employees to return to normal levels of functioning [33,34]. These needs are thus avoidance focused. 

Autonomy, mastery, meaning, and affiliation, on the other hand, focus on generating new resources that can be utilized in the future and thus constitute approach needs. Characterized by the need to experience ownership of one’s behavior, autonomy is one of the basic psychological needs proposed by Deci and Ryan [14]. Mastery focuses on seeking out new learning opportunities and optimal challenges to experience feelings of achievement and competence [32]. Meaning refers to participating in activities that add a sense of purpose and value to people´s lives [35]. Finally, affiliation is characterized by the human desire to experience belonging with other people [14]. Employees may craft their jobs and leisure time to satisfy these domain-independent psychological needs. 

While these six needs are indeed considered fairly universal across people and life domains (and satisfaction of these needs is universally beneficial in terms of well-being), people may vary in the importance they attach to each need [21,36]. The Needs Model of Crafting proposes that, depending on which needs are more salient to employees, they will engage in particular, need-congruent crafting efforts that primarily satisfy these needs. For example, two employees perceiving that their need to learn new things at work is not satisfied may differ in terms of the importance they attach to mastery need satisfaction, which in turn, may either motivate or inhibit their crafting efforts for mastery. In this study, we will first investigate this key proposition for both job and off-job crafting effort. This means that we anticipate motives for detachment from work, relaxation, autonomy, mastery, meaning, and affiliation (i.e., DRAMMA) to explain employees’ respective need-congruent job and off-job crafting efforts [37]. 

We expect that the psychological needs will also lead to need-congruent crafting efforts during leisure time because research has demonstrated that crafting is not limited to work. Crafting can transition between life domains, and people can likewise benefit from proactively adjusting their leisure [38,39] and other non-work time activities [40]. Proactive involvement in the non-work domain may actually play an important role in furthering one’s sustainable career across the lifespan through the generation of important resources which can be used across various life domains [41,42]. Indeed, Kelly and colleagues [42] recently showed that engaging in leisure activities can support employees’ sustainable careers by providing them with additional psychological resources that are also relevant in the work domain.

Active engagement in leisure activities has been shown to play an important role in enhancing employees’ well-being [43,44] and leisure crafting was proposed as a strategy through which people proactively shape their leisure activities to experience enjoyment and meaning in life [38]. Petrou and Bakker [39] showed that weekly leisure crafting was associated with satisfaction of the need for autonomy and relatedness. Employees crafted their leisure more when they experienced stress at work, but also when they had more autonomy at home, enabling them to engage in leisure crafting efforts. Scholars have more recently expanded the definition of leisure crafting to include not only recreational leisure activities (e.g., sports, hobbies), but also other non-work time activities (e.g., childcare, domestic tasks) that people may actively adjust during their non-work time (e.g., “home crafting”) [40]. A recent study demonstrated that increases in Finnish and Japanese employees’ attempts to craft their off-job time to satisfy their needs for meaning and affiliation were associated with feeling more energized [45]. Moreover, Kosenkranius and colleagues [46] recently developed an off-job crafting intervention to enhance employees’ needs-based off-job crafting efforts to increase their well-being.

According to the conservation of resources (COR) theory [47], people have the motivation to obtain, retain and protect resources. Shi and colleagues [48] showed that employees’ job crafting efforts can help to obtain and protect their resources during the workday which positively predicted their after-work energy levels. During off-job time, less or even no job demands are present and people are able to freely make decisions regarding their off-job time. Accordingly, people may have more opportunities to engage in crafting during off-job time than during work time. Therefore, in addition to investigating whether employees’ psychological needs indeed drive need-congruent job crafting, we explore whether these needs also motivate them to make need-congruent off-job crafting efforts. We hypothesize:

**Hypothesis** **1:** *Employees’ psychological needs (i.e., detachment, relaxation, autonomy, mastery, meaning, affiliation) are positively associated with need-congruent job and off-job crafting.*

### 2.2. Focus on Opportunities at Work as a Motivational Antecedent of Crafting

While all employees can potentially craft to experience psychological needs satisfaction [10], underlying individual differences, motivational and job characteristics and the social context may either enhance or inhibit employees’ crafting efforts [16,30]. So far, research has mainly investigated the role of stable individual characteristics, such as proactive personality, Big Five personality traits and regulatory focus as antecedents of job crafting [16,30]. Motivational antecedents which may vary across time and circumstances have received far less attention [25]. Uncovering the role of these more flexible antecedents of crafting can provide a more detailed understanding of employees’ efforts to shape their behaviors to their own benefit.

The literature further suggests that engagement in proactive behaviors (such as crafting) is a goal-driven process possibly with future-oriented thinking as one of its antecedents [20]. Carstensen and colleagues [49] proposed that perceived time left in life plays an important role in motivation and the processes of goal selection and pursuit. According to socioemotional selectivity theory (SST) [18,19], the goals people select depend on the time they perceive they have left in life. Based on the conceptualizations of general future time perspective [49,50], Zacher and Frese [17] adapted the construct to the work context, proposing that an occupational future time perspective consists of two conceptually different dimensions, namely employees’ perceptions of how much remaining time and remaining opportunities they (perceive they) have left in their occupational future. As employees are expected to retire at a certain age, perceived remaining time has been found to be more strongly linked to employees’ chronological age, while the negative relationship between chronological age and perceived future opportunities appears to be weaker and more complex, suggesting that additional person and work characteristics may influence the employees’ perceived future opportunities [17,50,51]. 

Following this line of reasoning, focus on opportunities at work, a sub-dimension of occupational future time perspective (OFTP) [17] is of particular interest in crafting. Focus on opportunities at work refers to the extent to which employees believe in new goals and opportunities in their occupational future. Interestingly, an occupational future time perspective has been shown to be more flexible across time and contexts than personality traits and is affected by individual characteristics such as age and physical health status [17,52]. Focus on opportunities at work may thus provide both employees and organizations with opportunities to shape behaviors and enhance well-being [25,53].

According to socioemotional selectivity theory [49], people who perceive their future as limited focus more on short-term goals that are emotionally meaningful to them and less on future-oriented goals, such as learning and development goals that could benefit them in the more distant future. Conversely, employees who perceive that they have more future opportunities at work may set goals and make plans that are long-term-oriented. An open time perspective may thus inspire employees’ approach crafting (i.e., crafting strategies that require investment of resources; crafting for autonomy, mastery, meaning and affiliation) to achieve their long-term goals. Kooij and colleagues [25] actually showed that particularly individuals with a more open-ended future perspective engage in job crafting strategies that promote future growth and development, as this is in line with their long-term goals. Their crafting efforts, in turn, were associated with higher work engagement and job performance [25]. A recent meta-analysis [54] further summarizes various positive links between focus on opportunities at work and different well-being and performance outcomes, thereby demonstrating the importance of the construct to both employees and organizations. Summing up, we believe that crafting is the behavioral mechanism which translates focus on opportunities into beneficial well-being outcomes. 

In light of this notion, we expect employees’ higher FoO at work to translate into engagement in job and off-job crafting for autonomy, mastery, meaning and affiliation (i.e., approach crafting). Contrastingly, because of their stronger future orientation, employees with high FoO could be more willing to invest energy and resources in achieving their goals, and therefore less eager to conserve their current resources and to avoid further resource losses. This could lead to less crafting for detachment from work and relaxation (i.e., avoidance crafting). Accordingly, we hypothesize:

**Hypothesis** **2a–b:** *Focus on opportunities at work is negatively associated with job and off-job crafting efforts for (a) detachment and (b) relaxation (avoidance crafting).*

**Hypothesis** **2c–f:** *Focus on opportunities at work is positively associated with job and off-job crafting efforts for (c) autonomy, (d) mastery, (e) meaning, and (f) affiliation (approach crafting).*

### 2.3. Psychological Needs as Mediators between Focus on Opportunities at Work and Crafting Efforts

FoO at work as a form of future-oriented thinking generates autonomous motivation to engage in proactive behaviors such as crafting to promote future growth and development [20]. This autonomous motivation is expressed in the form of concrete crafting motives that consequently lead to crafting efforts. As research so far has shown that employees’ open-ended future time perspective was associated with increases in long-term growth and development motives [55,56], we propose that the four approach needs of the DRAMMA model (i.e., autonomy, mastery, meaning, affiliation) positively mediate the relationship between FoO at work and crafting efforts targeting that specific need. These four needs promote positive gains in one’s future. Therefore, we expect that people who are optimistic about their future opportunities at work are more motivated to engage in crafting efforts for the satisfaction of these psychological needs.

Future-oriented goals are more distant in time and more resources are needed to achieve them, requiring future-oriented employees to invest more in important long-term goals over short-term gains [20]. Therefore, future-oriented employees’ motivation to satisfy their needs for detachment from work and relaxation could be weaker because they are willing to invest their current resources to achieve long-term goals. This means that employees with stronger FoO at work will have weaker avoidance needs (i.e., detachment, relaxation), consequently leading to less avoidance crafting. 

Besides focus on opportunities at work potentially leading to job crafting efforts via psychological needs, we also expect people to craft in the non-work domain. De Bloom and colleagues [13] proposed that employees who perceive satisfaction of a certain psychological need to be important to them may be inclined not only to attempt to engage in behaviors to satisfy that need in one domain, but may also engage in needs-based crafting efforts in other life domains. Therefore, we expect that focus on opportunities at work will also lead to off-job crafting efforts through psychological needs. Taken together, we hypothesize:

**Hypothesis** **3a–b:** *Needs (a) for detachment and (b) for relaxation (i.e., avoidance needs) mediate the negative relationship between focus on opportunities at work and need-congruent job and off-job crafting efforts.*

**Hypothesis** **3c–f:** *Needs (c) for autonomy, (d) for mastery, (e) for meaning and (d) affiliation (i.e., approach needs) mediate the positive relationship between focus on opportunities at work and need-congruent job and off-job crafting efforts.*

## 3. Methods

### 3.1. Participants and Procedure

We collected data in a three-wave survey study during 2018 and 2019 in Finland. This data collection was part of a larger research project. Finland has a high level of unionization, a strong social welfare system and work regulations in place, regulating employees working hours and allowing employees to establish a balance between their work and non-work time [57]. Additionally, Finnish employees have one of the smallest gender gaps in hours spent on care duties and housework in the EU, providing all employees with opportunities to engage in leisure activities [58]. Moreover, Finnish employees on average report high job autonomy and opportunities for personal growth at work which can promote and support employees crafting efforts also at work [59].

Participants completed three online surveys in Qualtrics with two three-month intervals between measurements of which we only use selected variables from the first two waves in this study. For recruitment, the research team contacted various public organizations and trade unions in Finland. Interested employees could sign up for the study by providing their contact information. The link to the first online survey was sent to all interested participants by email. Before starting to complete the survey, participants had to first confirm that they had read and understood the information concerning the purpose of the study, confidentiality, voluntary participation and data protection provided by the research team and agree to participate in this study. 

The original dataset consisted of 578 participants, but 48 participants did not complete the baseline questionnaire at T1 and were therefore excluded from further analyses, resulting in a sample size of 530. We conducted IBM SPSS Missing Values Analysis to understand the nature of missing values on all of our study variables. The response rate for variables of interest was 88.5–100% at T1 and 67.9–71.3% at T2. Little’s MCAR test was not significant (*χ*^2^ (158) = 167.18, *p* = 0.293), indicating that the missing data were randomly missing. After listwise deletion, the final dataset consisted of 346 participants without missing values on the key study variables. 

The final sample consisted of employees from various occupational fields (e.g., healthcare and social services, public administration, education). Participants’ ages ranged from 24 to 67 years (*M* = 48.8, *SD* = 10.0) and the sample mainly consisted of female participants (85.3%). Half of the participants (51.7%) had a university degree. Employees’ tenure in the organization varied from less than one year up to 42 years with the average being 15 years (*SD* = 11.4). The majority had a permanent work contract (87.0%) and on average, participants worked 38.9 h a week (*SD* = 4.5). 

### 3.2. Measures

All questionnaires were administered in Finnish language. Employees reported their focus on opportunities at work, psychological needs and proactive personality at the first measurement occasion (T1) and their off-job and job crafting efforts at the second measurement (T2). Background demographic data were recorded at the first measurement. Whenever available, we used translations of the scales validated in Finnish samples. For the scales without available translations, we hired professional translators to translate and back-translate the scale. Moreover, two native speakers and experts in the field also checked the translations. 

*Focus on opportunities at work* was assessed with a four-item scale [60]. An example item is: “My occupational future is filled with possibilities”. The scale ranged from 1 (does not apply at all) to 5 (applies completely). 

*Psychological needs* were assessed with a 10-item measure adapted from Chen and colleagues’ need valuation scale [36]. The items were adapted by the research team to correspond to the previously validated six-factor structure of the DRAMMA model [61]. Participants replied to one item per dimension for detachment (i.e., “It is important to me to mentally disengage from my work during my off-job time”) and relaxation (i.e., “It is important to me to relax after my work is done”) and two items per dimension for autonomy (e.g., “It is important to me to determine my own course of action”), mastery (e.g., “It is important to me to develop my skills and abilities”), meaning (e.g., “It is important to me to experience meaning in my life”), and affiliation (e.g., “It is important to me to experience close connections to the people around me”). Participants indicated the perceived importance of each need on a scale from 1 (not important at all) to 5 (very important). 

*Off-job crafting efforts* were measured with a new 18-item off-job crafting scale (blinded for review). Off-job crafting for each of the six DRAMMA dimensions was measured with three items. Example items are: “Over the past month…”, “…I’ve arranged my off-job time so that I distance myself from work-related tasks” (detachment), “…I’ve made sure to experience relaxation of my body and mind during off-job time” (relaxation), “…I’ve organized my off-job activities so that I determine my own course of action” (autonomy), “… I’ve organized my off-job activities so that I put my skills, knowledge or abilities into action” (mastery), “…I’ve made sure to experience meaning in my life during off-job time” (meaning), and “…I’ve made sure to experience close connections to the people around me during off-job time” (affiliation). Participants were asked to indicate the frequency of their engagement in off-job crafting over the past month on a scale from 1 (never) to 5 (very often). 

*Job crafting efforts* were assessed with a new 18-item scale (blinded for review) that mirrors the six-factor structure of the DRAMMA model. Each dimension was measured with three items and the example items are: “Over the past month…”, “…I’ve organized my work so that I switch off from work duties during off-job time” (detachment), “…I’ve arranged my work so that I get some rest during off-job time” (relaxation), “…I´ve made sure the things I do at work reflect what I really want in my job” (autonomy), “…I’ve arranged my work so that I experience proficiency in the things I undertake” (mastery), “…I’ve organized my work so that I achieve a sense of purpose in what I am doing” (meaning), and “…I’ve planned my work so that I feel relatedness in my work” (affiliation). Participants indicated how often they had engaged in job crafting over the past month on a scale from 1 (never) to 5 (very often). 

### 3.3. Control Variables

We included several control variables. We controlled for employees’ proactive personality to ensure that their crafting efforts are specifically driven by focus on opportunities at work and not by employees’ general tendency to behave proactively across time and circumstances. Proactive personality was assessed with a 6-item version of the Proactive Personality Scale [62]. An example item is: “If I see something I don’t like, I fix it” and employees indicated their agreement with the statements on a scale from 1 (totally disagree) to 5 (totally agree). Additionally, we controlled for employees’ chronological age, because FoO in one’s occupational future has often been shown to be negatively associated with chronological age [17,52]. In general, older employees are expected to have fewer future opportunities at work than younger employees due to situational limitations and loss of personal resources that employees may face due to aging [17,60]. Therefore, participants were asked to report their year of birth at the first measurement to enable us to calculate their ages in years. 

### 3.4. Statistical Analyses

First, we conducted diagnostic analyses to examine common method bias (CMB) and construct distinctiveness. Since all data were self-reported, we examined the potential presence of CMB by using Harman’s single-factor test [63]. The unrotated factor solution showed that the first factor accounted for only 26.8% of the variance. This is less than the 50% threshold, suggesting that the common method variance was not a substantial concern in our study.

To establish discriminant validity of our constructs, we calculated the square roots of the average variance extracted (AVE) of each construct which was assessed with more than one item and compared it with the shared variance between the constructs [64,65]. The AVE estimate for FoO at work was 0.79 and the estimates for need motives ranged from 0.50 to 0.79, for off-job crafting from 0.37 to 0.84 and for job crafting from 0.48 to 0.78. Meanwhile, the squared factor-level correlations between our constructs ranged from 0.00 to 0.40. This means, the AVE estimate for each construct was greater than its shared variance with any other constructs, indicating discriminant validity of our constructs.

We used confirmatory factor analysis (CFA) to test the fit of six-factor DRAMMA model of off-job crafting and job crafting scales. We compared the six-factor models with one-factor crafting models where all items loaded on the same factor and two-factor models where detachment from work and relaxation (avoidance crafting) were treated as one factor and autonomy, mastery, meaning and affiliation (approach crafting) as another factor. The analyses were performed using IBM SPSS Amos 25.0 (IBM Corp., Armonk, NY, USA) [66]. The estimation method was maximum likelihood. The goodness-of-fit of crafting models was evaluated by using the following indices: comparative fit index (CFI), Tucker–Lewis Index (TLI), root mean square error of approximation (RMSEA), and standardized root mean square residual (SRMR). Following the recommendations of Schermelleh-Engel and colleagues [67], for a good model fit, CFI and TLI values should be above 0.97, and RMSEA and SRMR under 0.05. For an acceptable model fit, CFI and TLI values are expected to be greater than 0.95, RMSEA between 0.05 and 0.08, and SRMR between 0.05 and 0.10.

### 3.5. Hypotheses Testing

We tested the hypotheses using IBM SPSS Statistics version 25 (IBM Corp., Armonk, NY, USA) [68] with Hayes’ PROCESS macro, model 4 [69,70]. As the DRAMMA-model [22] proposes that each of the six mechanisms can uniquely promote well-being, we treated each need as a separate construct and conducted separate analyses for each matching psychological need and crafting effort (i.e., need-congruent crafting). Following the procedure of Preacher and Hayes [70], we conducted mediation analyses for twelve simple mediation models. In each model, we entered FoO at work as the independent variable, one of the six psychological needs as the mediator and a respective corresponding job crafting or off-job crafting dimension as the dependent variable. In all our analyses, we controlled for chronological age and proactive personality. Following the recommendations of Hayes [69], we used a bootstrapping method with 5000 samples to test our indirect effects. The indirect effect was considered significant when the bootstrapped 95% confidence interval around the unstandardized indirect effect did not include zero.

## 4. Results

### 4.1. Descriptive Statistics

Table 1 shows the means, standard deviations, Cronbach’s alpha coefficients and bivariate correlations for all study variables. We found that FoO at work was positively correlated with autonomy, mastery, meaning and affiliation needs, off-job crafting for mastery, meaning and affiliation and job crafting for autonomy, mastery, meaning and affiliation. Each need was also positively associated with its corresponding off-job crafting dimension and job crafting dimension. Chronological age was negatively associated with mastery need and positively associated with off-job crafting for relaxation, autonomy, mastery and meaning, and job crafting for relaxation and affiliation. Proactive personality was positively correlated with autonomy, mastery and meaning needs, off-job crafting for mastery and meaning and job crafting for autonomy, mastery, meaning and affiliation. 

The results of the confirmatory factor analyses for both off-job crafting and job crafting scales are presented in Table 2. The hypothesized six-factor model indicated an acceptable fit for the job crafting scale, *χ*^2^ (115) = 319.57, CFI = 0.96, TLI = 0.94, RMSEA = 0.07, SRMR = 0.06. The six-factor model had the best fit to the data compared to a one-factor model of job crafting and a two-factor model, where JC for detachment and relaxation loaded on one factor and JC for autonomy, mastery, meaning and affiliation on another factor. The six-factor model of off-job crafting showed good fit, *χ*^2^ (115) = 209.43, CFI = 0.98, TLI = 0.97, RMSEA = 0.05, SRMR = 0.05. The model showed better fit than a one-factor model of off-job crafting and a two-factor model where OJC for detachment and relaxation loaded on one factor and OJC for autonomy, mastery, meaning and affiliation on another factor.

### 4.2. Hypotheses Testing

As shown in Table 3, all six needs at T1 were positively associated with employees’ job and off-job crafting efforts for the corresponding need at T2 after controlling for chronological age, proactive personality and FoO at work. This lends support to Hypothesis 1. After controlling for chronological age and proactive personality, we found that FoO at work at T1 was positively related to job crafting efforts for autonomy, mastery, meaning and affiliation and off-job crafting efforts for mastery, meaning, and affiliation at T2, thereby lending partial support to Hypothesis 2c–f. 

**Figure 1:**
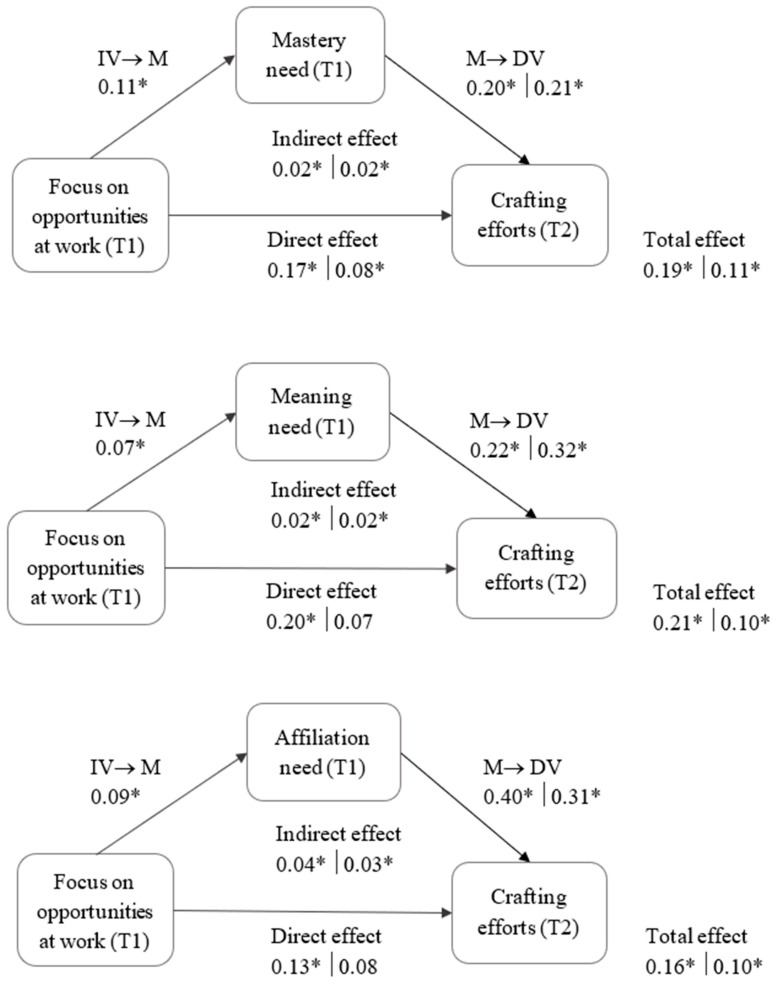
Significant mediation models depicting relationships between FoO at work, psychological needs, job and off-job crafting efforts. Note: relationships for JC on the left side of the vertical bar and for OJC on the right side. Significant effects are marked with *.

We tested the indirect effect of FoO at work on employees’ job and off-job crafting efforts through the psychological needs while controlling for chronological age and proactive personality at T1. The results of all simple mediation analyses with either job crafting or off-job crafting as the outcome are presented in Table 3 and in Figure 1. We found a significant indirect effect between FoO work and mastery, meaning and affiliation job crafting efforts through the corresponding needs. Furthermore, we found that mastery, meaning and affiliation needs also had a positive mediating role in the relationship between FoO at work and off-job crafting to satisfy these needs. The analyses did not reveal significant indirect effects in terms of detachment, relaxation and autonomy needs (H3a–c) neither in terms of job nor off-job crafting. Additionally, we found that the direct paths between FoO at work and job crafting for autonomy, mastery, meaning and affiliation remained significant. For off-job crafting, mastery was the only dimension on which the direct path between FoO and off-job crafting remained significant. Taken together, Hypothesis 3d–f regarding mastery, meaning and affiliation received support. 

## 5. Discussion

The aim of this study was to elucidate the role of employees’ focus on opportunities at work and psychological needs in their off-job and job crafting efforts and to test whether the relationships between FoO at work and different job and off-job crafting efforts are mediated by the six DRAMMA needs [22]. We tested mediations by making optimal use of the time lags in our dataset, separating independent and dependent variables in time and we controlled for chronological age and proactive personality traits.

Our preparatory analyses showed that proactive personality was positively associated with approach crafting for all needs in the job domain and mastery and meaning needs in the off-job domain, supporting the notion that employees who are generally more proactive are more likely to engage in crafting [16,39]. Chronological age, a construct closely linked to FoO at work showed positive associations with some crafting dimensions. Chronological age appeared to be associated more strongly to the off-job crafting dimensions than job crafting dimensions. This suggests that older employees engage in more diverse crafting efforts than younger employees during their non-work time. 

Integrating the focus on opportunities at work concept to crafting [17], we found support for positive associations between FoO at work and approach-oriented crafting efforts, namely job crafting efforts for autonomy, mastery, meaning and affiliation and off-job crafting efforts for mastery, meaning and affiliation. 

In line with the existing research on growth motives acting as mediators between future time perspective and job crafting [25,55,56], we found that mastery, meaning and affiliation needs mediated the relationship between FoO at work and job crafting efforts. Furthermore, our results revealed similar positive mediation relationships in terms of off-job crafting for mastery, meaning and affiliation, supporting the notion that psychological needs are not domain-specific and can indeed lead to crafting efforts in different life domains [13]. It appears that higher FoO at work leads to stronger mastery, meaning and affiliation needs which, in turn, motivates employees to engage in crafting efforts both in the work and in the non-work domain. These approach-oriented crafting efforts could generate new resources that employees can use across life domains.

Contrary to our expectations, FoO at work was not negatively associated with job and off-job crafting efforts for detachment and relaxation (avoidance crafting), nor did these needs, as well as the autonomy need, mediate the relationship between FoO and job/off-job crafting. As detachment from work and relaxation are avoidance needs, we expected that employees who focus on their occupational future opportunities would not be specifically motivated to organize their work and leisure time in a way that helps them to preserve their current resources and prevent further resource losses. Instead, they would be more invested in such crafting efforts that may require additional effort but also maximize potential long-term gains. Focusing on their long-term goals may therefore not enhance their motivation to engage in crafting efforts to experience detachment from work and relaxation [71]. However, detachment from work and relaxation are indispensable to everyone’s optimal functioning, as they both allow employees to handle various already existing demands and contribute to recovery from stressful events [33,34]. Therefore, while not being of highest importance for future-oriented employees, avoidance crafting might still be practiced at some level by everyone to maintain their optimal functioning and well-being.

Furthermore, we found no support for the hypothesis that autonomy need acts as a mediator between FoO at work and job and off-job crafting efforts. Autonomy need mediated the relationship between FoO at work and job and off-job crafting efforts after controlling for chronological age. However, this mediation effect disappeared after taking into account the effects of employees’ proactive personality. This finding is consistent with the notion that employees with proactive personality are more likely to take the initiative and search for new opportunities, to more readily take action and to strive for their goals even when facing challenges [62,72]. As proactive personality entails acting autonomously in various circumstances, FoO at work may not be a necessary antecedent to stronger autonomy needs. Employees with proactive personality traits are already constantly more willing to take the initiative and seek out new opportunities in different environments [62].

In accordance with the propositions of the new Needs Model of Crafting [13], we found that all six needs were positively associated with the corresponding dimensions of job and off-job crafting efforts. Employees who reported stronger motivation to satisfy a particular psychological need engaged more in job and off-crafting efforts related to that need. 

### 5.1. Theoretical Implications

This study contributes to the crafting literature by examining the relationships between employees’ focus on opportunities at work, variation in employees’ psychological needs and crafting efforts in different life domains. Our main aim was to integrate the FoO at work concept [17] and needs theories to predict crafting behaviors and put propositions of the newly developed Needs Model of Crafting to the test. 

First, our empirical findings about FoO at work being positively associated with job crafting for autonomy, and with both job and off-job crafting for mastery, meaning and affiliation advance our knowledge about the relationship between FoO at work and proactive crafting efforts in different life domains. These findings are in line with those of earlier research proposing that FoO at work is an important motivational antecedent of proactive behaviors allowing employees to generate resources needed to achieve their future work-related goals [20,73]. 

Crafting efforts such as planning one’s work time to improve skills or arranging one’s private life to experience more meaning require investment of energetic resources. However, in return for these efforts, employees will potentially gain new resources that can consequently help them in their future careers. FoO at work may therefore be a resource that motivates people to engage in crafting efforts, resulting in accumulative resource gains referred to as resource caravans [74]. This is also in line with the conservation of resources theory, stating that people who have gained more resources are in a better position to invest these resources in order to gain further resources [75]. Additionally, our findings are in line with those of earlier research showing that individuals with more open-ended futures are more likely to engage in crafting efforts that promote future growth and development [25,55]. Therefore, we contribute to the crafting literature by demonstrating that FoO at work is an important motivational resource for employees of all ages to engage in approach-oriented job and off-job crafting.

Second, to the best of our knowledge, this is the first study to examine all six DRAMMA needs [22] as employees’ crafting motives. Building on earlier crafting theories that have recognized psychological needs as job crafting motives [10] and the Needs Model of Crafting [13] that emphasizes the importance of employee’s motives for crafting efforts, we contribute to the knowledge of the psychological mechanisms that can act as motivational antecedents of both job and off-job crafting. Our findings suggest that employees with higher needs for detachment, relaxation, autonomy, mastery, meaning or affiliation engage more in need-congruent crafting efforts. By examining each psychological need separately, we showed the unique relationship of each need with need-congruent crafting efforts, demonstrating that people are indeed motivated to engage in crafting efforts that target satisfaction of specific, personally important psychological needs. Moreover, we found evidence that psychological needs can motivate crafting efforts in both work and non-work domains. These findings are in line with those of earlier research about psychological needs as motivators for people to engage in goal-directed behaviors that can lead to need satisfaction in different life domains [21,28]. While earlier crafting literature has proposed and shown that crafting efforts are related to satisfaction of psychological needs [76], we showed that specific needs can motivate subsequent need-congruent crafting efforts in both life domains, thereby testing important propositions of De Bloom and colleagues’ Needs Model of Crafting [13]. This model states that psychological needs serve both as drivers and rewards of crafting efforts.

Third, we contribute to the crafting literature by recognizing employees’ opportunities to engage in needs-based crafting in different life domains. We demonstrated that mastery, meaning and affiliation needs not only mediate the relationship between FoO at work and job crafting efforts, but also motivate employees to seek out ways to craft their off-job time, resulting in potential new resources that can in turn be utilized in the work domain [38,42]. Our findings are in line with the work of Kooij and colleagues [25,55,56] in the job domain and extend the existing empirical knowledge about the associations between future time perspective, growth motives and job crafting, also to the non-work domain. 

### 5.2. Practical Implications

Our findings have important implications to both employees and organizations by underlining the importance of FoO at work and psychological needs as motivational antecedents of engaging in proactive behaviors both at work and during leisure time. Our findings show that variations in employees’ perceived FoO at work and their psychological needs can affect their engagement in various crafting efforts, which, in turn, can potentially lead to higher performance and better well-being outcomes (e.g., organizational citizenship behaviors, work engagement). Essentially, the results inform organizations who may benefit most from crafting training, and on how such training should look like. 

Our findings also emphasize the importance of considering employees’ crafting efforts in the non-work domain as higher FoO at work and psychological needs lead to higher engagement in off-job crafting, potentially allowing employees to gain resources that are later beneficial in the work domain. Therefore, not only could sufficient high-quality leisure time enhance the well-being and performance of an employee, but it could also help organizations to retain more proactive, motivated and high-performing employees. Our findings also indicate that when it comes to designing organizational crafting interventions, we should look beyond the “one size fits all” approach and offer more flexible interventions that accommodate employee’s individual needs to maximize the gains from such interventions.

Importantly, while potential resource gains from crafting efforts could result in positive gain spirals for employees who already have more FoO at work, it may also leave employees with limited FoO in a disadvantaged position. Lack of FoO at work could lead to further resource loss circles [75,77], potentially leading to even lower engagement in crafting efforts. These employees particularly could benefit from organizational support. While job and off-job crafting are seen as bottom-up approaches, organizations can still support employees in their crafting efforts by creating conditions that can increase employees’ FoO at work. As FoO at work is a flexible construct, organizations could increase their employees’ FoO at work by providing them with information about learning opportunities and different career trajectories within the organization and by assisting employees with their planning and long-term goal setting [52,78,79]. This way, organizations can create environments where employees are encouraged to explore their future opportunities to motivate them to engage in crafting efforts that can assist them in attaining their future goals. 

One employee group that could benefit especially from both time-broadening and crafting interventions is older employees. Namely, crafting efforts that are motivated by employees’ FoO at work and intrinsic psychological needs may contribute to sustainable careers among the aging workforce [77]. Older employees are generally expected to have lower FoO at work than younger employees because of potential age-related resource losses [17,60]. Assisting older employees in exploring their occupational opportunities to increase their crafting efforts could help older employees to manage the potential age-related losses more successfully and prolong their careers. 

### 5.3. Crafting and the COVID-19 Pandemic

Since the start of the pandemic, research on crafting as a tool to cope with the consequences of the crisis has been flourishing. The first theoretical [80], qualitative [81] and quantitative evidence [82,83] does indeed suggest that crafting work, leisure and the boundaries between life domains has the potential to buffer against the negative effects of the crisis on mental health and provide workers with a sense of meaning. However, the remote working context also poses new challenges and opportunities in motivating people to craft. On the one hand, autonomy increases and makes it easier to craft some aspects of one´s job and leisure time (e.g., integrate exercise or napping into the working day). On the other hand, social contacts are more limited, potentially leading workers to pursue other (i.e., need-congruent) crafting efforts than before. It is also more difficult for organizations and leaders to reach their employees to encourage them to engage in crafting behaviors and to increase their focus on opportunities. Structural, targeted face-to-face meetings may be required to inspire and train people to use crafting efforts to acquire a future time perspective on their work, to craft, to reflect on and to proactively address their psychological needs. 

### 5.4. Limitations and Future Research 

The present study is not without limitations. First, while the sample consisted of employees from various professions, over two thirds of the participants came from the fields of healthcare and social services, public administration and education and the majority of the participants were female, potentially limiting the generalizability of our findings to other populations of workers. Moreover, the majority of the participants were full-time knowledge workers with permanent work contracts. Future research should further explore the relationships between FoO at work, needs and crafting efforts among employees with less traditional work conditions, such as freelancers and entrepreneurs, professions where being oriented to future opportunities and proactive crafting efforts may be especially relevant.

Second, while our study adopted a holistic perspective on crafting in different life domains and although we utilized parsimonious measurement of crafting at work and outside the work domain, our measurement only included occupational focus on opportunities. As employees are assumed to hold various role identities in different life domains, it could be useful for future research to also investigate employees’ perceived opportunities in particular non-work domains such as hobbies or volunteer work. Doing so would further enhance our understanding of how employees can benefit from experiences in one domain in some other domain. Third, in our study, we expect employees’ crafting efforts to lead to needs satisfaction and positive outcomes in terms of regeneration of resources and better well-being in different life domains. However, we did not directly test this proposition. Future research should investigate whether need-congruent crafting efforts do indeed result in these positive outcomes. Fourth, we only measured employees’ FoO at work at T1 and, therefore, we cannot rule out the reverse causality of the relationship between FoO and crafting efforts. It is possible that besides higher FoO at work motivating employees in their crafting efforts, employees’ proactive engagement in crafting efforts could subsequently create more future opportunities at work. 

Fifth, our research focuses on employees’ individual crafting efforts as an individual-level phenomenon, without considering their social and organizational context. However, employees’ social relations and collaborative goals could potentially either hinder or stimulate employees’ crafting opportunities and motivation for attaining certain psychological needs both at work and during off-job time. For example, there is empirical evidence for collective crafting which refers to a group´s joint efforts to change the nature of their work environment (e.g., [84,85,86]). Such collective crafting is, in turn, supported or hindered by team- or organizational-level processes. Future research could therefore consider how these social dynamics affect crafting across the different life domains. More intensive longitudinal designs, such as experience sampling methods that integrates data from other social players (e.g., family members, friends, colleague), could provide detailed insights into these relationships. 

Sixth, our study was conducted in Finland where employees have high autonomy to influence both their work and leisure time. Because of this unique sociocultural context, our findings might not be generalizable to other sociocultural contexts. Future studies could consider different sociocultural contexts and cross-cultural differences when investigating employees’ needs-based crafting efforts (for first insights on cross-cultural differences in crafting, see for instance [87,88,89]).

## 6. Conclusions

In this study, we focused on elucidating the role of motivational drivers of job and off-job crafting efforts to better understand what guides employees in their crafting efforts. The present study contributes to the existing literature by enhancing our knowledge about focus on opportunities at work and six psychological needs as the motivational drivers of need-congruent crafting efforts in different life domains. We found that employees with greater perceived future opportunities at work attached greater importance to mastery, meaning and affiliation needs (i.e., approach needs), which in turn lead to higher engagement in need-congruent job and off-job crafting efforts. In addition, we found that all six DRAMMA needs were positively associated with both forms of crafting efforts (i.e., job crafting and off-job crafting). FoO at work and psychological needs can therefore explain some of the hypothesized crafting patterns both at work and during non-work time. We hope that our holistic approach to crafting will inspire future research on need-congruent crafting in various life domains and provide both employees and organizations with helpful insights into fostering employees’ crafting efforts to enhance and support sustainable work lives. 

## Figures and Tables

**Table 1:** Means, standard deviations and correlations of study variables.

Variables	M	SD	1	2	3	4	5	6	7	8	9	10	11	12	13	14	15	16	17	18	19	20	21
1. Age (T1)	48.8	10.0																					
2. Proactive personality (T1)	3.7	0.7	−0.03	(0.81)																			
3. FoO at work (T1)	2.9	1.2	−0.35 **	0.29 **	(0.94)																		
4. Detachment need (T1)	4.3	0.9	−0.05	0.00	−0.07																		
5. Relaxation need (T1)	3.9	0.9	−0.02	−0.06	−0.04	0.49 **																	
6. Autonomy need (T1)	4.4	0.6	−0.04	0.26 **	0.15 **	0.24 **	0.32 **	(0.67)															
7. Mastery need (T1)	4.3	0.6	−0.13 *	0.30 **	0.29 **	0.13 *	0.19 **	0.46 **	(0.66)														
8. Meaning need (T1)	4.5	0.5	−0.03	0.17 **	0.19 **	0.19 **	0.25 **	0.40 **	0.59 **	(0.71)													
9. Affiliation need (T1)	4.2	0.8	0.00	0.10	0.14 *	0.15 **	0.18 **	0.25 **	0.30 **	0.44 **	(0.88)												
10. OJC for detachment (T2)	3.6	1.0	0.04	−0.01	−0.02	0.29 **	0.17 **	0.11 *	0.11 *	0.13 *	0.06	(0.91)											
11. OJC for relaxation (T2)	3.5	0.8	0.18 **	0.04	−0.02	0.17 **	0.19 **	0.14 **	0.13 *	0.09	0.09	0.59 **	(0.84)										
12. OJC for autonomy (T2)	3.8	0.8	0.23 **	0.03	−0.02	0.10	0.17 **	0.21 **	0.03	0.07	0.09	0.39 **	0.58 **	(0.86)									
13. OJC for mastery (T2)	3.3	0.7	0.15 **	0.27 **	0.17 **	0.01	0.02	0.20 **	0.25 **	0.18 **	0.16 **	0.40 **	0.42 **	0.46 **	(0.67)								
14. OJC for meaning (T2)	3.7	0.8	0.14 **	0.17 **	0.11 *	0.06	0.13 *	0.26 **	0.20 **	0.24 **	0.21 **	0.44 **	0.49 **	0.54 **	0.65 **	(0.89)							
15. OJC for affiliation (T2)	3.8	0.8	0.07	0.08	0.12 *	0.10	0.10	0.13 **	0.19 **	0.18 **	0.31 **	0.35 **	0.41 **	0.40 **	0.38 **	0.55 **	(0.87)						
16. JC for detachment (T2)	3.7	0.9	0.06	−0.03	−0.02	0.25 **	0.22 **	0.15 **	0.06	0.10	0.15 **	0.63 **	0.50 **	0.42 **	0.34 **	0.40 **	0.36 **	(0.91)					
17. JC for relaxation (T2)	3.6	1.0	0.14 **	0.01	0.00	0.09	0.15 **	0.14 *	0.07	0.07	0.14 **	0.50 **	0.61 **	0.50 **	0.43 **	0.46 **	0.39 **	0.79 **	(0.92)				
18. JC for autonomy (T2)	3.4	0.9	0.10	0.24 **	0.25 **	−0.04	0.04	0.19 **	0.17 **	0.06	0.06	0.29 **	0.34 **	0.36 **	0.35 **	0.38 **	0.37 **	0.41 **	0.54 **	(0.84)			
19. JC for mastery (T2)	3.7	0.7	0.05	0.24 **	0.30 **	−0.03	0.00	0.19 **	0.26 **	0.19 **	0.15 **	0.29 **	0.27 **	0.25 **	0.51 **	0.41 **	0.38 **	0.37 **	0.45 **	0.58 **	(0.76)		
20. JC for meaning (T2)	3.7	0.8	0.08	0.21 **	0.27 **	−0.06	0.02	0.15 **	0.22 **	0.22 **	0.23 **	0.21 **	0.24 **	0.28 **	0.43 **	0.47 **	0.39 **	0.33 **	0.40 **	0.58 **	0.69 **	(0.86)	
21. JC for affiliation (T2)	3.4	0.9	0.15 **	0.13 *	0.16 **	0.05	0.12 *	0.14 **	0.15 **	0.20 **	0.38 **	0.17 **	0.26 **	0.24 **	0.33 **	0.35 **	0.42 **	0.31 **	0.36 **	0.37 **	0.47 **	0.61 **	(0.89)

Note: *n* = 346; FoO = focus on opportunities, JC = job crafting; OJC = off-job crafting, T1 = baseline, T2 = 3 months. Cronbach’s alphas appear along the diagonal in brackets. * *p* < 0.05; ** *p* < 0.01.

**Table 2:** Model comparison for confirmatory factor analyses.

Model	*χ* ^2^	*df*	CFI	TLI	RMSEA	SRMR
Six-factor JC model	319.57	115	0.96	0.94	0.07	0.06
Two-factor JC model	428.01	127	0.94	0.92	0.08	0.07
One-factor JC model	600.91	117	0.90	0.87	0.11	0.10
Six-factor OJC model	209.43	115	0.98	0.97	0.05	0.05
Two-factor OJC model	358.41	123	0.89	0.94	0.08	0.10
One-factor OJC model	358.89	122	0.94	0.93	0.08	0.07

Note: JC = job crafting; OJC = off-job crafting.

**Table 3:** Model coefficients for twelve separate simple mediation analyses.

	DRAMMA Dimension					
	Detachment	Relaxation	Autonomy	Mastery	Meaning	Affiliation
X → M	*b* = −0.08, SE = 0.05, 95% CI [−0.18, 0.01]	*b* = −0.03, SE = 0.04, 95% CI [−0.12, 0.06]	*b* = 0.04, SE = 0.03, 95% CI [−0.02, 0.09]	*b* = 0.11, SE = 0.03, 95% CI [0.05, 0.16]	*b* = 0.07, SE = 0.03, 95% CI [0.02, 0.13]	*b* = 0.09, SE = 0.04, 95% CI [0.01, 0.17]
Job crafting						
M → Y	*b* = 0.26, SE = 0.05, 95% CI [0.16, 0.37]	*b* = 0.18, SE = 0.06, 95% CI [0.06, 0.29]	*b* = 0.20, SE = 0.08, 95% CI [0.04, 0.36]	*b* = 0.20, SE = 0.06, 95% CI [0.07, 0.32]	*b* = 0.22, SE = 0.08, 95% CI [0.07, 0.38]	*b* = 0.40, SE = 0.06, 95% CI [0.29, 0.51]
Total effect	*b* = 0.01, SE = 0.05, 95% CI [−0.09, 0.10]	*b* = 0.05, SE = 0.05, 95% CI [−0.05, 0.14]	*b* = 0.20, SE = 0.04, 95% CI [0.12, 0.29]	*b* = 0.19, SE = 0.03, 95% CI [0.12, 0.25]	*b* = 0.21, SE = 0.04, 95% CI [0.13, 0.29]	*b* = 0.16, SE = 0.04, 95% CI [0.08, 0.25]
Direct effect	*b* = 0.02, SE = 0.05, 95% CI [−0.07, 0.12]	*b* = 0.05, SE = 0.05, 95% CI [−0.06, 0.15]	*b* = 0.19, SE = 0.04, 95% CI [0.11, 0.28]	*b* = 0.17, SE = 0.03, 95% CI [0.10, 0.23]	*b* = 0.20, SE = 0.04, 95% CI [0.12, 0.27]	*b* = 0.13, SE = 0.04, 95% CI [0.04, 0.21]
Indirect effect	*b* = −0.02, SE = 0.01, 95% CI [−0.05, 0.00]	*b* = −0.01, SE = 0.01, 95% CI [−0.03, 0.01]	*b* = 0.01, SE = 0.01, 95% CI [−0.00, 0.02]	*b* = 0.02, SE = 0.01, 95% CI [0.00, 0.04]	*b* = 0.02, SE = 0.01, 95% CI [0.00, 0.04]	*b* = 0.04, SE = 0.02, 95% CI [0.00, 0.07]
Off-job crafting						
M → Y	*b* = 0.31, SE = 0.05, 95% CI [0.20, 0.41]	*b* = 0.19, SE = 0.05, 95% CI [0.10, 0.29]	*b* = 0.32, SE = 0.08, 95% CI [0.17, 0.47]	*b* = 0.21, SE = 0.06, 95% CI [0.09, 0.33]	*b* = 0.32, SE = 0.08, 95% CI [0.16, 0.47]	*b* = 0.31, SE =0.05, 95% CI [0.20, 0.42]
Total effect	*b* = −0.00, SE = 0.05, 95% CI [−0.10, 0.09]	*b* = 0.03, SE = 0.04, 95% CI [−0.06, 0.11]	*b* = 0.04, SE = 0.04, 95% CI [−0.04, 0.12]	*b* = 0.11, SE = 0.03, 95% CI [0.04, 0.17]	*b* = 0.10, SE = 0.04, 95% CI [0.02, 0.18]	*b* = 0.10, SE = 0.04, 95% CI [0.02, 0.19]
Direct effect	*b* = 0.02, SE = 0.05, 95% CI [−0.07, 0.12]	*b* = 0.03, SE = 0.04, 95% CI [−0.05, 0.11]	*b* = 0.03, SE = 0.04, 95% CI [−0.05, 0.11]	*b* = 0.08, SE = 0.03, 95% CI [0.02, 0.15]	*b* = 0.07, SE = 0.04, 95% CI [−0.01, 0.16]	*b* = 0.08, SE = 0.04, 95% CI [−0.00, 0.16]
Indirect effect	*b* = −0.03, SE = 0.02, 95% CI [−0.06, 0.00]	*b* = −0.01, SE = 0.01, 95% CI [−0.03, 0.01]	*b* = 0.01, SE = 0.01, 95% CI [−0.01, 0.03]	*b* = 0.02, SE = 0.01, 95% CI [0.01, 0.04]	*b* = 0.02, SE = 0.01, 95% CI [0.00, 0.05]	*b* = 0.03, SE = 0.01, 95% CI [0.00, 0.06]

Note: *n* = 346, X = focus on opportunities at work, M = psychological need, Y = job crafting or off-job crafting effort, CI = confidence interval. Unstandardized coefficients presented. Chronological age and proactive personality were entered as covariates. Bootstrap samples size = 5000.

## Data Availability

The data that support the findings of this study are available from the corresponding author upon reasonable request.
